# Foundation Models for Generative AI in Time-Series Forecasting

**DOI:** 10.2196/76964

**Published:** 2025-07-25

**Authors:** Diva Beltramin, Cedric Bousquet

**Affiliations:** 1 Medical Information Department Hospices Civils de Lyon Lyon France; 2 Laboratory of Medical Informatics and Knowledge Engineering in e-Health Sorbonne University Paris France; 3 Public Health Service and Medical Information Centre Hospitalier Universitaire de Saint-Étienne Saint-Etienne France

**Keywords:** large language models, LLM, foundation models, time series, generative artificial intelligence, artificial intelligence, electronic health records, electronic medical records, systematic reviews, disease trajectory, machine learning, algorithms, forecasting

We read with great interest the article entitled “Generative AI Models in Time-Varying Biomedical Data: Scoping Review” by He et al [1]. The authors performed a thorough and exhaustive review of generative artificial intelligence (AI) models used for the analysis of biomedical data varying over time. However, the foundation models (FMs) identified in this article do not seem to correspond to the proposed definition, which is “models capable of performing various generative tasks after being trained on extremely large and typically unlabeled datasets.” Additionally, the authors slightly modified Wornow et al’s [2] definition of FMs by adding the “generative” adjective to the word “tasks.” As a consequence, this definition should designate only FMs that support generative AI, but the authors mention some models that have no ability for generation.

Moreover, the authors propose two distinct FM lists in two different sections of the article. The first list features models for time-series forecasting, while the second includes large language models trained on electronic health records (referred to as clinical language models [CLaMs]) that take text as input and may produce text as output.

In the first list of FMs, the authors included generative adversarial networks (GANs), variational autoencoders, conditional variational autoencoders, omicsGAN, Potential Energy Underlying Single-Cell Gradients (PRESCIENT), gene-guided weakly supervised clustering via GANs, and trained GAN discriminator. However, these models are not FMs, as they were not trained on large amounts of data, and we are not aware of the possibility that they were pretrained to enable later fine-tuning on different downstream tasks.

In the second list, the authors erroneously mention several CLaMs as examples of FMs that support generative AI. Some of these models have been trained on tasks previously used to train bidirectional encoder representations from transformers, such as predicting a masked word based on previous and following words, and are known as masked language models. Indeed, masked language models may have text as input and output, as in tasks like summarization or question answering. However, these models are not generative AI models. For example, GatorTron [3], which is cited in the article, is a masked language model, but it has a generative AI version, GatorTronGPT [4].

Additionally, CLaMs are not naturally able to perform time-series forecasting and thus have to be repurposed or made capable of transforming the forecasting task into a language task to achieve the forecasting ability [5].

In conclusion, besides our concerns about FMs, we were favorably surprised by this article, which presents innovative applications of generative AI for those who are used to traditional biostatistics. Some applications of generative AI to time-varying biomedical data may be limited to specific niches where they are particularly efficient and not replaceable, and it is unclear if generative AI may outperform traditional time-series approaches. Nevertheless, it is advantageous to have the possibility to choose among several options when practicing data analysis, and this article shows the richness of the different methods available.

## Abbreviations

AI: artificial intelligence
CLaM: clinical language model
FM: foundational model
GAN: generative adversarial network
PRESCIENT: Potential Energy Underlying Single-Cell Gradients
